# Decision Support for Clinician Referral of Patients With Potential *BRCA1/2* Mutations for Genetic Counseling: A Secondary Analysis of a Cluster Randomized Clinical Trial

**DOI:** 10.1001/jamanetworkopen.2024.41175

**Published:** 2024-10-24

**Authors:** Rita Kukafka, Samuel Pan, Thomas Silverman, Wendy K. Chung, Mary Beth Terry, Elaine Fleck, Richard G. Younge, Jill Dimond, Katherine D. Crew

**Affiliations:** 1 Department of Biomedical Informatics, Vagelos College of Physicians and Surgeons, Columbia University Irving Medical Center, New York, New York; 2 Herbert Irving Comprehensive Cancer, Columbia University Irving Medical Center, New York, New York; 3 Department of Sociomedical Sciences, Mailman School of Public Health, Columbia University Irving Medical Center, New York, New York; 4 Department of Pediatrics and Medicine, Columbia University Irving Medical Center, New York, New York; 5 Department of Epidemiology, Columbia University Irving Medical Center, New York, New York; 6 Division of Community and Population Health, New York Presbyterian Hospital, New York, New York; 7 Sassafras Collective, Ann Arbor, Michigan; 8 Department of Medicine, Vagelos College of Physicians and Surgeons, Columbia University Irving Medical Center, New York, New York

## Abstract

This secondary analysis of a randomized clinical trial reports primary care clinician outcomes of decision support tools for referral of patients with potential *BRCA1/2* mutations for genetic counseling.

## Introduction

It is critical to develop targeted interventions to address barriers that impede appropriate *BRCA1/2* referrals to genetic counselors from primary care clinicians following US Preventive Services Task Force (USPSTF) guidelines.^1^ We conducted a cluster randomized clinical trial (Genomics for Breast Cancer in Primary Care [BEATRICE]) of patient and clinician decision support to increase *BRCA1/2* genetic referrals based on USPSTF guidelines. The study design, patient-level characteristics, and results have been published previously.^2,3^ Here, we report the effectiveness of the intervention on clinician outcomes.

## Methods

The Columbia University Irving Medical Center Institutional Review Board approved this secondary analysis of BEATRICE (NCT03470402); the trial protocol is provided in Supplement 1. Participants provided electronic informed consent. We followed the AAPOR reporting guideline.

Data were obtained from clinicians who completed baseline and 6-month surveys (eMethods in Supplement 2). Surveys assessed demographics, practice characteristics, and predictors of genetic counseling referral behavior (attitudes, subjective norms, and perceived behavior control), informed by the theory of planned behavior.^4^ Baseline surveys of clinicians who did not complete the 6-month survey were compared with those who did, with no significant differences. We examined frequency distributions of clinician baseline and 6-month survey outcomes and participant demographic characteristics. We compared both groups using χ^2^ tests for categorical variables and *t* tests (or Wilcoxon rank-sum tests when nonparametric) for continuous variables; 2-sided *P* < .05 was statistically significant. Analyses were performed using SAS, version 9.4 (SAS Institute).

## Results

Of 85 clinicians enrolled, 74 (87.0%) completed baseline and 6-month surveys and were included in this analysis. Clinicians (mean [SD] age, 43.1 [12.4] years) were diverse in terms of specialty and race and ethnicity (Table 1). Most were women (67 [90.5%] vs 7 men [9.5%]), and most (53 [71.6%]) practiced in community clinics serving predominantly Medicaid or Medicare patients. At baseline, 33 clinicians (44.6%) reported being moderately or very confident in counseling patients regarding inherited risk; 23 (31.1%) reported being moderately or very confident in discussing possible management options according to risk. With respect to subjective norms, having assessed patient risk for hereditary breast and ovarian cancer (HBOC), 52 clinicians (70.3%) reported that most other clinicians would expect them to make a referral to genetic testing services; 32 (43.2%) reported that patients would expect them to make a referral. Regarding perceived behavioral control, 24 clinicians (32.4%) reported that it would be very or somewhat difficult to refer patients to genetic testing services after assessing patient risk for HBOC (mean [SD] score, 4.4 [1.5]).

**Table 1.** Clinician Characteristics, Confidence, Subjective Norms, Attitudes, and Perceived Behavioral Control at Baseline^a^

Variable	All clinicians (N = 74)	Intervention group (n = 38)	Control group (n = 36)
**Characteristic**			
Age, mean (SD), y	43.1 (12.4)	45.4 (11.8)	40.7 (12.8)
Sex			
Male	7 (9.5)	6 (15.8)	1 (2.8)
Female	67 (90.5)	32 (84.2)	35 (97.2)
Race			
Asian	16 (21.6)	9 (23.7)	7 (19.4)
Black	10 (13.5)	6 (15.8)	4 (11.1)
White	46 (62.2)	22 (57.9)	24 (66.7)
Unknown	2 (2.7)	1 (2.6)	1 (2.8)
Ethnicity			
Hispanic or Latino	12 (16.2)	7 (18.4)	5 (13.9)
Not Hispanic or Latino	62 (83.8)	31 (81.6)	31 (86.1)
Specialty			
Family practice	11 (14.9)	7 (18.4)	4 (11.1)
Internal medicine	28 (37.8)	16 (42.1)	12 (33.3)
Obstetrics and gynecology	33 (44.6)	14 (36.8)	19 (52.8)
Other	2 (2.7)	1 (2.6)	1 (2.8)
Clinic setting^b^			
Private	21 (28.4)	11 (29.0)	10 (27.8)
Community	53 (71.6)	27 (71.1)	26 (72.2)
**Confidence in managing care of patients with a family history of breast or ovarian cancer (range, 0-4)^c^**			
Score, mean (SD)	2.0 (1.4)	2.0 (1.6)	2.1 (1.2)
Q1: Taking a brief family history and making a referral decision			
Not at all or somewhat confident (0)	14 (18.9)	12 (31.6)	2 (5.6)
Moderately or very confident (1)	60 (81.1)	26 (68.4)	34 (94.4)
Q2: Counseling patients regarding inherited risk			
Not at all or somewhat confident (0)	41 (55.4)	23 (60.5)	18 (50.0)
Moderately or very confident (1)	33 (44.6)	15 (39.5)	18 (50.0)
Q3: Discussing other risk factors for breast cancer			
Not at all or somewhat confident (0)	39 (52.7)	19 (50.0)	20 (55.6)
Moderately or very confident (1)	35 (47.3)	19 (50.0)	16 (44.4)
Q4: Discussing possible management options, according to risk			
Not at all or somewhat confident (0)	51 (68.9)	23 (60.5)	28 (77.8)
Moderately or very confident (1)	23 (31.1)	15 (39.5)	8 (22.2)
**Subjective norms (range, 1-7)** ^d^			
Score, mean (SD)	4.9 (1.2)	5.2 (1.1)	4.7 (1.1)
Q1: Having assessed a patient’s risk of HBOC, patients expect me to make a referral to genetic testing services.			
Strongly disagree, disagree, or somewhat disagree (1-3)	14 (18.9)	6 (15.8)	8 (22.2)
Neither agree nor disagree (4)	28 (37.8)	13 (34.2)	15 (41.7)
Somewhat agree, agree, or strongly agree (5-7)	32 (43.2)	19 (50.0)	13 (36.1)
Q2: Having assessed a patient’s risk of HBOC, most other clinicians would expect me to make a referral to genetic testing services.			
Strongly disagree, disagree, or somewhat disagree (1-3)	5 (1.4)	2 (5.2)	3 (8.3)
Neither agree nor disagree (4)	17 (23.0)	9 (23.7)	8 (22.2)
Somewhat agree, agree, or strongly agree (5-7)	52 (70.3)	27 (70.1)	25 (69.4)
**Perceived behavioral control (range, 1-7) (Q: Having assessed a patient’s risk of HBOC, making a referral to genetic testing services would be…)** ^d^			
Score, mean (SD)	4.4 (1.5)	4.4 (1.6)	4.5 (1.4)
Very or somewhat difficult (1-3)	24 (32.4)	11 (28.9)	13 (36.1)
Neither difficult nor easy (4)	15 (20.3)	11 (29.0)	4 (11.1)
Very or somewhat easy (5-7)	35 (47.3)	16 (42.1)	19 (52.8)
**Attitudes (range, 0-7**) **and knowledge (range, 0-5) (Q: Having assessed a patient’s risk of hereditary breast or ovarian cancer, making a referral decision based on current guidance is generally…)**			
Attitudes^e^			
Score, mean (SD)	6.0 (0.8)	6.0 (0.8)	6.0 (0.8)
Q1: Helpful or very healthful	48 (64.9)	26 (68.4)	22 (61.1)
Q2: Necessary or very necessary	57 (77.0)	30 (78.4)	27 (75.0)
Q3: Appropriate or very appropriate	65 (87.8)	32 (84.2)	33 (91.7)
Knowledge^f^			
Score, mean (SD)	4.0 (0.9)	3.9 (0.9)	4.1 (0.8)
Q1: Predictive genetic tests for *BRCA1/2* mutations are able to identify patients at high risk of developing breast cancer.			
Agree	71 (96.0)	36 (94.7)	35 (97.2)
Q2: Women with breast cancer and a strong family history should perform *BRCA1/BRCA2* testing.			
Agree	68 (91.9)	34 (89.5)	34 (94.4)
Q3: Scientific evidence recommends *BRCA1/BRCA2* positive women receive clinical and instrumental surveillance starting from the age of 25.			
Agree	42 (56.8)	24 (63.2)	50 (50.0)
Q4: The percentage of breast cancer cases associated with BRCA1/2 mutations is:			
15%-35% or >50%	27 (36.5)	17 (44.7)	10 (27.8)
1%-10%	47 (63.5)	21 (55.3)	26 (72.2)
Q5: The absolute risk of developing breast cancer in the presence of *BRCA1/2* mutations is:			
<10% or 100%	5 (6.8)	4 (10.5)	1 (2.8)
40%-80%	69 (93.2)	34 (89.5)	35 (97.2)

Abbreviations: HBOC, hereditary breast and ovarian cancer; Q, question.

^a^ Unless indicated otherwise, values are presented as No. (%) of clinicians.

^b^ Private clinic setting refers to the faculty practice at Columbia University Irving Medical Center, whereas community clinic setting refers to an affiliated network of ambulatory care settings with emphasis on providing services regardless of inability to pay and usually takes care of Medicaid or Medicare patients.

^c^ Confidence in management score (range, 0-4) is the sum of its 4 items.

^d^ Subjective norms and perceived behavioral control scores are the calculated means.

^e^ Attitude scale evaluates a clinician’s attitudes about a referral. A higher score indicates more favorable attitudes.

^f^ Knowledge score was assessed with 5 items. A higher number of correct answers indicates more accurate knowledge.

At 6 months, we found significant differences in clinician confidence in discussing possible management options according to patient risk (Table 2). Confidence was higher among the intervention vs control groups (13 [34.2%] vs 5 [13.9%] were moderately or very confident; *P* = .04). We also observed significant differences in subjective norms, with intervention clinicians more likely than control clinicians to agree or strongly agree with the patient’s expectation of making a referral to genetic testing services (mean [SD] score, 5.2 [1.1] vs 4.7 [1.1]; *P* = .02). We observed significant differences between the intervention vs control groups in perceived behavioral control to make a referral to genetic testing services after assessing patient risk for HBOC (mean [SD] score, 5.3 [1.5] vs 4.6 [4.6]; *P* = .02).

**Table 2.** Clinician Outcomes at 6 Months^a^

Variable	All clinicians (N = 74)	Intervention group (n = 38)	Control group (n = 36)	*P* value
**Confidence in managing care of patients with a family history of breast or ovarian cancer (range, 0-4)^b^**				
Score, mean (SD)	2.0 (1.4)	2.3 (1.5)	1.8 (1.3)	.08
Q1: Taking a brief family history and making a referral decision				
Not at all or somewhat confident (0)	13 (17.6)	7 (18.4)	6 (16.7)	.84
Moderately or very confident (1)	61 (82.4)	31 (81.6)	30 (83.3)	
Q2: Counseling patients regarding inherited risk				
Not at all or somewhat confident (0)	42 (56.8)	19 (50.0)	23 (63.9)	.23
Moderately or very confident (1)	32 (43.2)	19 (50.0)	13 (36.1)	
Q3: Discussing other risk factors for breast cancer				
Not at all or somewhat confident (0)	34 (45.9)	15 (39.5)	19 (52.8)	.25
Moderately or very confident (1)	40 (54.1)	23 (60.5)	17 (47.2)	
Q4: Discussing possible management options, according to risk				
Not at all or somewhat confident (0)	56 (75.7)	25 (65.8)	31 (86.1)	.04
Moderately or very confident (1)	18 (24.3)	13 (34.2)	5 (13.9)	
**Subjective norms (range, 1-7)^c^**				
Score, mean (SD)	4.9 (1.1)	5.2 (1.1)	4.7 (1.1)	.02
Q1: Having assessed a patient’s risk of HBOC, patients expect me to make a referral to genetic testing services (range, 1-7)				
Strongly disagree (1)	1 (1.4)	0	1 (2.8)	.04
Disagree (2)	5 (6.8)	4 (10.5)	1 (2.8)	
Somewhat disagree (3)	10 (13.5)	1 (2.6)	9 (25.0)	
Neither agree nor disagree (4)	17 (23.0)	7 (18.4)	10 (27.8)	
Somewhat agree (5)	8 (10.8)	6 (15.8)	2 (5.6)	
Agree (6)	31 (41.9)	19 (50.0)	12 (33.3)	
Strongly agree (7)	2 (2.7)	1 (2.6)	1 (2.8)	
Q2: Having assessed a patient’s risk of HBOC, most other clinicians would expect me to make a referral to genetic testing services (range, 1-7)				
Strongly disagree (1)	0	0	0	.60
Disagree (2)	3 (4.1)	1 (2.6)	2 (5.6)	
Somewhat disagree (3)	5 (6.8)	1 (2.6)	4 (11.1)	
Neither agree nor disagree (4)	14 (18.9)	7 (18.4)	7 (19.4)	
Somewhat agree (5)	16 (21.6)	8 (21.1)	8 (22.2)	
Agree (6)	29 (39.2)	16 (42.1)	13 (36.1)	
Strongly agree (7)	7 (9.5)	5 (13.2)	2 (5.6)	
**Perceived behavioral control (range, 1-7)**				
Score, mean (SD)	4.9 (1.1)	5.3 (1.5)	4.6 (1.4)	.02
**Behavioral intention (range, 1-7)**				
Score, mean (SD)	6.1 (0.9)	6.3 (0.8)	6.0 (1.0)	.12
**Knowledge (range, 0-5)** ^d^				
Score, mean (SD)	4.2 (0.8)	4.3 (0.7)	4.1 (0.8)	.22
**Attitudes (range, 1-7)^e^**				
Score, mean (SD)	6.2 (0.6)	6.2 (0.6)	6.2 (0.6)	.47

Abbreviations: HBOC, hereditary breast and ovarian cancer; Q, question.

^a^ Unless indicated otherwise, values are presented as No. (%) of clinicians.

^b^ Confidence in management score is the sum of 4 items.

^c^ Subjective norms score is assessed by calculating the mean of Q1 and Q2.

^d^ Knowledge score for HBOC is assessed with 5 items. A higher number of correct answers indicates increased knowledge.

^e^ Attitude scale evaluates a clinician’s attitudes about a referral. A higher score indicates more favorable attitudes.

## Discussion

These findings suggest that patient and clinician decision support could affect clinician outcomes (including confidence, perceived behavioral control, and subjective norms) that are associated with behavioral intention and enactment.^4^ The implications of these findings for designing interventions support the need to target theoretically informed barriers, moving beyond clinician education alone, which has been shown to have minimal effect on clinician adoption of clinical genetic practices.^5^ Study limitations include recruitment from a single institution and the moderate sample size; additionally, larger system-level barriers were not examined, and the self-reported data may be limited by response options. Additional studies are needed to examine the effectiveness of theoretically informed decision support interventions in the context of referral decision-making to genetic counseling services.

## Notes

Supplement 1.
Trial Protocol

Supplement 2.
eMethods

eReferences

Supplement 3.
Data Sharing Statement
