# Supplementary material for: Decision Support for Clinician Referral of Patients With Potential BRCA1/2 Mutations for Genetic Counseling: A Secondary Analysis of a Cluster Randomized Clinical Trial
Source: JAMA Netw Open. 2024 Oct 24;7(10):e2441175. doi: 10.1001/jamanetworkopen.2024.41175 (PMC11581495; doi:10.1001/jamanetworkopen.2024.41175)
Supplement: Supplement 2. — eMethods eReferences [file jamanetwopen-e2441175-s002.pdf]

Supplementary Online Content

Kukafka R, Pan S, Silverman T, et al. Decision support for clinician referral of patients with potential *BRCA1/2* mutations for genetic counseling: a secondary analysis of a cluster randomized clinical trial. *JAMA Netw Open*. 2024;7(10):e2441175. doi:10.1001/jamanetworkopen.2024.41175

eMethods
eReferences

This supplementary material has been provided by the authors to give readers additional information about their work.

© 2024 Kukafka R et al. *JAMA Network Open.*

eMethods

In the analysis presented in this paper, we were interested in examining clinician-level outcomes associated with the (BEATRICE, Genomics for Breast Cancer in Primary Care), a cluster randomized controlled trial (RCT) of patient and clinician decision support designed to increase *BRCA1/2* genetic referrals based on U.S. Preventive Services Task Force (USPSTF) guidelines. BEATRICE trial enrollment began in December 2018, and follow-up survey data collection ended in August 2020. The study design, patient-level characteristics, and results have previously been published.[1]

Recruitment of the Clinicians

The clinicians were from Columbia University Irving Medical Center (CUIMC) outpatient clinics in New York City who had consented to the BEATRICE trial. To be eligible, a clinician must be a physician, nurse practitioner, physician assistant, or nurse-midwife affiliated with clinics associated with the Columbia University Irving Medical Center (CUIMC). They were approached in person, via mail, email, or phone.

Clinician Consent

Clinicians were given the option to review the consent form online through Qualtrics, which recorded the clinician's consent to participate. The clinician was also given the option to schedule a time to complete a verbal consent over the phone or an in-person appointment where written consent may be documented. For clinicians who consented by phone, the research staff fully discussed the informed consent form with the clinician, and verbal consent was obtained. Clinicians who consented in person were presented with the same consent forms, and their signatures were obtained. Clinicians were encouraged to ask questions, and it explained that they

© 2024 Kukafka R et al. *JAMA Network Open.*

could withdraw at any time. Clinician participants were recruited because they are involved in the clinical encounter and care of the patients who are enrolled in the study.

Clinician Compensation:

The clinician participant received $50 for the baseline survey and $50 for the 6-month follow-up survey. The Columbia University Irving Medical Center (CUIMC) institutional review board approved the study procedures.

Clinician Survey Measures

Surveys were administered to clinicians at baseline and 6 months after randomization. The clinician measures included demographic and professional/practice characteristics, subjective risk communication confidence[2], confidence in managing patients with a family history of breast or ovarian cancer[3], genetic testing knowledge[4], attitudes[5], subjective norms[6], perceived behavioral control[6], and behavioral intention[6]. *Subjective risk communication confidence*[2] was assessed using three questions ascertaining clinicians' perceived level of confidence in their knowledge of medical statistics, ability to communicate medical statistics to patients and/or families effectively, and ability to help patients and/or families understand information about probabilities or risks. *Confidence in managing patients with a family history of breast or ovarian cancer*[3] was assessed in four aspects of managing patients with a family history of breast/ovarian cancer, taking a brief family history and making a referral decision, counseling patients regarding inherited risk, discussing other risk factors for breast cancer, and discussing possible management options according to risk. *Knowledge about predictive genetic testing for HBOC*[4] was investigated through four questions using a three-point options Likert scale ("agree," "uncertain," and "disagree"). A fifth question assessed understanding of the absolute risk of developing breast cancer in the presence of *BRCA1/2* PV. *Attitudes* in the

© 2024 Kukafka R et al. *JAMA Network Open.*

context of *BRCA1/2 referral*[5] were assessed using a single item that asked clinicians to rate how helpful-unhelpful, necessary-unnecessary, and appropriate-inappropriate making a referral decision based on current guidance would be after assessing a patient's risk of HBOC. Items to assess *subjective norms*[6] (perceived pressure from others to do or not to do the behavior), *perceived behavioral control*[6] (beliefs about factors likely to facilitate or inhibit the behavior), and *behavioral intention* to make a referral[6] were derived from those developed for previous studies[6] and focused on a single behavior, referral to genetic testing services after assessing a patient's risk for HBOC.

Sample Size and Completion

The primary outcome measure and analysis plan for the main trial determined the sample size.[1,7] Eighty-five (85) clinicians were recruited to enroll 190 patients in the parent BEATRICE study. Of the 85 clinicians enrolled in the BEATRICE study, 74 (87%) completed the baseline and 6-month surveys and are included in this analysis.

Clinician Recruitment and Survey Completion Flow Diagram

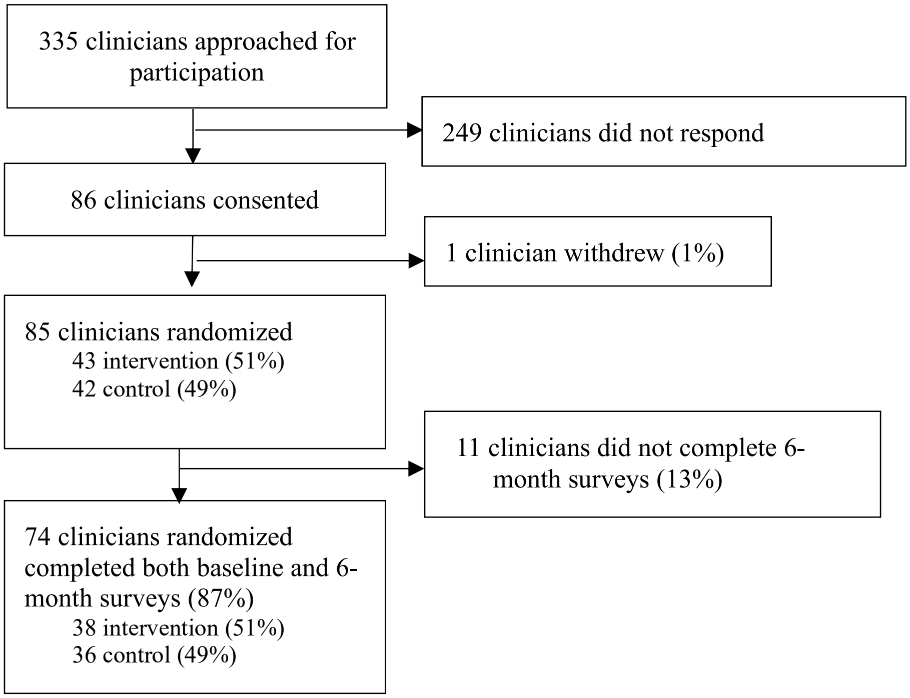

© 2024 Kukafka R et al. *JAMA Network Open.*

eReferences

1. Kukafka, R., Pan, S., Silverman, T., Zhang, T., Chung, W.K., Terry, M.B., Fleck, E., Younge, R.G., Trivedi, M.S., McGuinness, J.E., et al. (2022). Patient and Clinician Decision Support to Increase Genetic Counseling for Hereditary Breast and Ovarian Cancer Syndrome in Primary Care: A Cluster Randomized Clinical Trial. JAMA Netw Open *5*, e2222092. 10.1001/jamanetworkopen.2022.22092.
2. Han, P.K.J., Joekes, K., Elwyn, G., Mazor, K.M., Thomson, R., Sedgwick, P., Ibison, J., and Wong, J.B. (2014). Development and evaluation of a risk communication curriculum for medical students. Patient Education and Counseling *94*, 43-49. http://dx.doi.org/10.1016/j.pec.2013.09.009.
3. Watson, E., Clements, A., Yudkin, P., Rose, P., Bukach, C., Mackay, J., Lucassen, A., and Austoker, J. (2001). Evaluation of the impact of two educational interventions on GP management of familial breast/ovarian cancer cases: a cluster randomised controlled trial. Br J Gen Pract *51*, 817-821.
4. Marzuillo, C., De Vito, C., Boccia, S., D'Addario, M., D'Andrea, E., Santini, P., Boccia, A., and Villari, P. (2013). Knowledge, attitudes and behavior of physicians regarding predictive genetic tests for breast and colorectal cancer. Preventive medicine *57*, 477-482.
5. Bouhnik, A.-D., N'Diaye, K., Evans, D.G., Harris, H., Tibben, A., van Asperen, C., Schmidtke, J., Nippert, I., Mancini, J., and Julian-Reynier, C. (2017). Validation of a scale for assessing attitudes towards outcomes of genetic cancer testing among primary care providers and breast specialists. PloS one *12*, e0178447.
6. Wilson, B.J., Islam, R., Francis, J.J., Grimshaw, J.M., Permaul, J.A., Allanson, J.E., Blaine, S., Graham, I.D., Meschino, W.S., and Ramsay, C.R. (2016). Supporting genetics in primary care: investigating how theory can inform professional education. European Journal of Human Genetics *24*, 1541.
7. Silverman, T.B., Vanegas, A., Marte, A., Mata, J., Sin, M., Ramirez, J.C.R., Tsai, W.Y., Crew, K.D., and Kukafka, R. (2018). Study protocol: a cluster randomized controlled trial of web-based decision support tools for increasing BRCA1/2 genetic counseling referral in primary care. BMC Health Serv Res *18*, 633. 10.1186/s12913-018-3442-x.

© 2024 Kukafka R et al. *JAMA Network Open.*
