# Supplementary material for: Decision Support for Clinician Referral of Patients With Potential BRCA1/2 Mutations for Genetic Counseling: A Secondary Analysis of a Cluster Randomized Clinical Trial
Source: JAMA Netw Open. 2024 Oct 24;7(10):e2441175. doi: 10.1001/jamanetworkopen.2024.41175 (PMC11581495; doi:10.1001/jamanetworkopen.2024.41175)
Supplement: Supplement 3. — Data Sharing Statement [file jamanetwopen-e2441175-s003.pdf]

Data Sharing Statement

Kukafka. Decision Support for Clinician Referral of Patients With Potential BRCA1/2 Mutations for Genetic Counseling. *JAMA Netw Open*. Published October 24, 2024.
doi:10.1001/jamanetworkopen.2024.41175

Data
Data available: Yes
Data types: Deidentified participant data
How to access data: Data requests to Rita Kukafka rk326@cumc.columbia.edu
When available: With publication

Supporting Documents
Document types: None

Additional Information
Who can access the data: Data will be made available for researchers whose proposed use of the data has been approved with a signed data access agreement.
Types of analyses: For a specified purpose.
Mechanisms of data availability: With a signed data access agreement.
Any additional restrictions: no additional restrictions.
